# Contracting as a bridging factor linking outer and inner contexts during EBP implementation and sustainment: a prospective study across multiple U.S. public sector service systems

**DOI:** 10.1186/s13012-020-00999-9

**Published:** 2020-06-11

**Authors:** Rebecca Lengnick-Hall, Cathleen Willging, Michael Hurlburt, Karissa Fenwick, Gregory A. Aarons

**Affiliations:** 1 The Brown School, Washington University, St. Louis, MO USA; 2 Pacific Institute for Research and Evaluation, Albuquerque, NM USA; 3 Suzanne Dworak-Peck School of Social Work, University of Southern California, Los Angeles, CA USA; 4 VA Greater Los Angeles Healthcare System, Los Angeles, CA USA; 5 Department of Psychiatry, University of California, San Diego, La Jolla, CA USA; 6 UC San Diego Dissemination and Implementation Science Center (UC San Diego-DISC), La Jolla, CA USA

**Keywords:** EPIS framework, Open system, Bridging factors, Resource dependence, Outer context, Inner context, Public sector, Service systems, Evidence-based practice

## Abstract

**Background:**

Bridging factors are relational ties (e.g. partnerships), formal arrangements (e.g. contracts or polices) and processes (e.g. data sharing agreements) linking outer and inner contexts and are a recent evolution of the Exploration-Preparation-Implementation-Sustainment (EPIS) framework. Bridging factor research can elucidate ways that service systems may influence and/or be influenced by organizations providing health services. This study used the EPIS framework and open systems and resource dependence theoretical approaches to examine contracting arrangements in U.S. public sector systems. Contracting arrangements function as bridging factors through which systems communicate, interact, and exchange resources with the organizations operating within them.

**Methods:**

The sample included 17 community-based organizations in eight service systems.

Longitudinal data is derived from 113 contract documents and 88 qualitative interviews and focus groups involving system and organizational stakeholders. Analyses consisted of a document review using content analysis and focused coding of transcripts from the interviews and focus groups. A multiple case study analysis was conducted to identify patterns across service systems and organizations. The dataset represented service systems that had sustained the same EBP for between 2 and 10 years, which allowed for observation of bridging factors and outer-inner context interactions over time.

**Results:**

Service systems and organizations influenced each other in a number of ways through contracting arrangements. Service systems influenced organizations when contracting arrangements resulted in changes to organizational functioning, required organizational responses to insufficient funding, and altered interorganizational network relationships. Organizations influenced service systems when contract arrangements prompted organization-driven contract negotiation/tailoring, changes to system-level processes, and interorganizational collaboration. Service systems and organizations were dependent on each other as implementation progressed. Resources beyond funding emerged, including adequate numbers of eligible clients, expertise in the evidence-based practice, and training and coaching capacity.

**Conclusion:**

This study advances implementation science by expanding the range and definition of bridging factors and illustrating specific bi-directional influences between outer context service systems and inner context organizations. This study also identifies bi-directional dependencies over the course of implementation and sustainment. An analysis of influence, dependencies, and resources exchanged through bridging factors has direct implications for selecting and tailoring implementation strategies, especially those that require system-level coordination and change.

Contributions to literature

- This study deepens understanding of bridging factors as a key issue for implementation science and illuminates a specific methodological approach for studying them.
- This study illustrates an open-system approach to evidence-based practice implementation and describes specific ways that systems and organizations influence and depend on each other over time.
- These findings can inform selection of system-level implementation strategies that implicate policy and funding arrangements.
- Explicit attention to outer-inner influences and resource dependencies when selecting and tailoring strategies can bring to light environmental constraints as well as available and needed resources that affect the potential success of implementation strategies in a particular service system.

## Background

### Bridging factors across the inner and outer context boundary

A key feature of commonly used implementation science conceptual frameworks such as the Exploration-Preparation-Implementation-Sustainment (EPIS), Consolidated Framework for Implementation Research, and Practical, Robust Implementation and Sustainability Model is a distinction between the outer and inner contexts/settings in which implementation of evidence-based practices (EBPs) occurs [1–4]. In the EPIS framework, the outer context encompasses factors outside of the organization. Examples are federal and state legislative priorities and policies, local service systems, funding arrangements, and interorganizational networks [2]. The EPIS inner context captures factors inside of the organization including culture/climate, leadership, individual characteristics, and processes such as fidelity monitoring and supervision [2].

To date, implementation researchers have focused more attention on the inner context [5]. In Novins et al.’s systematic review of dissemination and implementation empirical studies in children’s mental health, 92% of the studies examined inner context factors, while 37% examined outer context factors (overlap due to studies that reported both) [6]. In Moullin and colleagues’ 2019 systematic review of the EPIS framework, 90% of the projects examined inner context factors, while 57% examined outer context factors [1].

When outer and inner contexts are examined in the same study, researchers often separately examine and report features of outer and inner contexts, without explicitly accounting for the interdependence and bi-directional influences between service system and organizations within the service system. Scholars have separately reported outer and inner context factors in a variety of studies and settings [7–11]. As a result, little attention is paid to the ways outer context environments shape internal organizational functioning, or how organizations influence external environments during EBP implementation.

### An opportunity to expand organizational implementation research

Organizational theory can address both outer and inner contexts [12]. However, examining organizations without acknowledging their interconnectedness to the service system and other organizations in the system operates under the theoretical assumption that organizations are closed systems. In contrast, open-system perspectives highlight interdependence between outer and inner contexts [13, 14] and view organizations as part of a broader interdependent system that may range from simple to complex, rigid to flexible, and loosely to tightly coupled [14]. The degree of interdependence between system parts and the type of system flows (of materials, information, etc.) also vary [14] and can affect EBP implementation. An open-system approach considers the dynamics and interactions that occur within an organization’s relational web and is consistent with the EPIS framework’s concepts of “bridging factors” and interorganizational networks [1].

### Advancing the concept of bridging factors

Bridging factors are defined as “factors that cross or link the outer system and inner organizational context” [1]. Three examples provided by Moullin et al. [1] are community-academic partnerships, purveyors/intermediaries, and interagency collaboration, but the full range and impact of bridging factors have yet to be identified and described. Community-academic partnerships may bridge the outer and inner contexts by engaging multiple levels of stakeholders (e.g., system leaders, organizational leaders, academic partners, and frontline staff) [1]. Purveyors/intermediaries of an EBP may bridge outer and inner contexts by helping make adaptations that address both system and organizational needs and constraints [1]. Interagency collaboration may inherently involve outer-inner bridging through formal resource sharing agreements, system-wide EBP coaching [15] and training across multiple organizations, or multi-agency implementation teams.

Like the social network concept of boundary spanning, developed by Burt and others e.g., [16–20], bridging factors serve a specific function. Boundary spanners are network positions/roles that close the structural holes (i.e., network gaps) between individual actors or groups [21]. Closing structural holes facilitates communication, increases knowledge flow, enhances coordination, resolves conflict, and increases social capital within the broader network [21–23]. Boundary spanners may be educational outreach workers, academic detailers, knowledge brokers, opinion leaders, facilitators, or even teams [24–26]. We propose that bridging factors (1) may be relational ties (e.g., boundary spanners or community-academic partnerships) but may also refer to formal arrangements (e.g., contracts or policies) and processes (e.g., data sharing agreements), and (2) they serve a particular function within a bounded system that is implementing an EBP, namely, (3) they help or hinder implementation and sustainment by connecting or disconnecting the outer and inner contexts.

Studying bridging factors advances implementation science by directly addressing and clarifying dynamic outer-inner context boundaries that system and organizational leaders and staff continually confront when implementing and sustaining an EBP. For example, organizational level implementation can be subject to changing policies and funding that can affect inner context organization functioning [27]. Bridging factor research can elucidate ways that organizations are both influenced by and influence service systems. Enhanced understanding of bridging factors can help implementers plan, select, and tailor multilevel implementation strategies that proactively acknowledge and leverage bi-directional influences. Understanding the dynamic interrelationships between outer and inner contexts is also crucial to EBP sustainment [28–30].

This study focuses on one type of bridging factor—contracting arrangements that support the implementation of a specific EBP in multiple service systems. We chose contracting arrangements because they are the most common way for public entities to structure how community-based organizations deliver human services [31, 32]. Although this study takes place in the USA, contracting out public health services occurs in a variety of settings. For example, examining contracting arrangements as a potential bridging factor may also be applicable to the National Health Service systems that have privatized services, including those in the UK, Denmark, Finland, Norway, Sweden, Portugal, and Spain [33–37]. Contracting out governmental health services to non-governmental providers has also been examined in low- and middle-income countries such as Cambodia and Guatemala [38].

Through contracts, system administrators specify services, set eligibility requirements, determine billing and outcome reporting processes, delineate the number of type of clients that can be referred to a particular program, and decide the conditions under which organizations will get paid for services [27, 31, 39]. Contracting out services requires interorganizational collaboration between the public agency and the organizations that deliver services [31, 40].

Therefore, contracting arrangements represent a bridging factor because they function as mediums through which public sector systems communicate, interact, and exchange resources with the organizations that operate within them. Resource dependence theory emphasizes that organizations are inextricably tied to the environment(s) in which they operate and organizational survival depends on acquiring and maintaining essential resources [41]. Additionally, this theory draws attention to ways that organizations react to existing and changing environmental contingencies and constraints [41]. Resource dependence theory can help explain why and the ways in which organizational leaders acquire resources, form relationships, maintain autonomy, and manage dependence on other actors during EBP implementation [42, 43].

### Study context

The present study examined the implementation and sustainment of SafeCare®, a well-established EBP aimed at reducing and preventing child neglect [44, 45]. SafeCare is highly structured yet flexible, focusing on three content modules for improving parent skills in parent-child/infant interactions, home safety, and child health [46]. Services are provided in the family’s home. Home visitors, coaches, and trainers achieve and maintain certification, and coaches work closely with home visitors to ensure ongoing adherence to the SafeCare model [46].

The data for this study were drawn from larger mixed-method studies of EBP sustainment in 11 service systems. EBP implementation in public sector systems is typically funded through a combination of federal, state, and local sources [47]. Furthermore, a variety of systems, such as public health, mental health, human services, and justice, may be involved in the contracting arrangements that fund a specific implementation effort. This study examined one state and seven county-based child welfare systems that utilized both cost reimbursement and performance-based contracting arrangements to fund SafeCare implementation. While existing studies peripherally acknowledge the importance of contracts in EBP implementation and sustainment [48, 49], the question remains: How do organizations and systems interact around these contracting arrangements? The goal of the present study is to use an open-system theoretical approach in order to identify specific bi-directional and outer-inner context interactions and dependencies that shape EBP implementation and sustainment.

## Methods

### Study sample

The present study utilized contracting documents and secondary qualitative data collected during three prospective mixed-methods parent studies. The parent studies were conducted between 2008–2013, 2011–2015, and 2012–2017 and built upon a program of SafeCare effectiveness and implementation research that began in 2005. Inclusion criteria for the systems in this sample were that each system: (1) achieved SafeCare sustainment status, (2) was enrolled in the 2012–2017 NIMH funded sustainment-focused parent study (Grant# R01MH072961), and (3) had at least two time points for the secondary qualitative data (drawn from 2008-2013 and 2011-2015 projects). Service systems that did not fully sustain SafeCare were excluded.

Sustainment status was assessed by Aarons and colleagues [49] using criteria based on Stirman et al.’s [50] systematic review. Systems achieved SafeCare sustainment if core elements of the intervention were “maintained or delivered at a sufficient level of fidelity after initial implementation support has been withdrawn, and adequate capacity exists to continue maintaining these core elements” [49, 50]. The sample was drawn from one state-operated and seven county-operated child welfare systems. Embedded within these eight service systems were 17 community-based organizations (CBOs) contracted to provide SafeCare and other home visitation services by the state and county governments. Table 1 describes the population, income, geographical information, and year that SafeCare started for each of the eight service systems in this sample.

**Table 1:** Service system descriptive information

Service System	Population Estimate^a,b^	Median Household Income^a^	Persons in poverty^a^ (%)	Population per square mile^c^	Land in square miles^c^	Year system started SafeCare
State	3,943,079	$49,767	15.8	54.7	68,595	2003
County 1	1,419,516	$71,535	14.5	4,020.4	325	2008
County 2	464,493	$44,871	24.0	91.1	4824	2009
County 3	2,423,266	$60,807	12.9	303.8	7206	2012
County 4	884,363	$96,265	10.1	17,179.1	47	2011
County 5	448,150	$68,023	14.2	155.0	2735	2010
County 6	179,921	$47,258	17.0	46.9	3775	2009
County 7	854,223	$81,972	9.5	446.7	1843	2012

^a^2018 U.S. Census Bureau data

^b^2017 U.S. Census Bureau data

^c^2010 U.S. Census Bureau data

### Data sources

Data sources are described in Table 2. RL reviewed 113 SafeCare-related contract documents (e.g., statements of work). The research team collected 16 of the contracting documents during the parent studies, and the first author collected an additional 97 contracting documents through publicly available records, and/or communications (September–December 2018) with appropriate child welfare and/or contracting department personnel in each system.

**Table 2:** Data sources for each service system

Service system	Date range of collected data	# of organizations	Contracting documents		Qualitative data		
			# of documents	# of pages	Individual interviews	Small group interviews	Focus groups
State	2005–2016	3	21	606	19	6	0
County 1	2008–2018	4	17	1759	20	1	0
County 2	2009–2018	3	13	242	7	1	1
County 3	2011–2018	1	18	274	4	2	1
County 4	2011–2018	2	12	161	9	0	0
County 5	2010–2018	2	15	419	5	1	0
County 6	2009––2018	1	12	403	6	0	0
County 7	2012–2018	1	5	275	3	1	1
**Total**		**17**	**113**	**4,139**	**73**	**12**	**3**

The secondary qualitative dataset included 88 transcripts of 73 individual interviews, 12 small-group interviews, and three focus groups. Interviews and focus groups were tailored to the participants’ roles and explored SafeCare sustainment processes. Because this study examined service system and organizational interactions around contracting arrangements, data reflected perspectives of state or county level child welfare system personnel (e.g., system leaders), and agency leaders (e.g., executive directors) or other key members of the organization’s upper management who were involved in the contracting processes. If available, SafeCare coordinators and academic partners were included because they participated in contracting processes or were aware of contracting arrangements. SafeCare home visitors, coaches, and lower-level management were excluded because they were not involved or had little to no in-depth knowledge of contracting processes. There were 66 (unduplicated) participants including 33 state and county (“service system”) personnel, 30 agency leaders and members of upper management, one SafeCare coordinator, and two academic partners. In the parent studies, all interviews and focus groups were digitally recorded, professionally transcribed, de-identified, and checked for accuracy by at least one of the data collectors.

### Data analysis

Data analysis occurred in three stages: (1) a content analysis of contracting documents, (2) focused coding of transcripts to identify examples of bi-directional service system and organizational influence around these contracting arrangements, and (3) multiple case study analysis across the eight service systems. In the first analytic stage, a content analysis was completed following the steps outlined by Bernard and colleagues [51]. The goal of the content analysis was to identify specific ways that service systems directed SafeCare implementation in the CBOs via contracting arrangements. RL completed a line-by-line analysis of each document. Codes documented details of SafeCare implementation including service delivery (e.g., caseload size), staffing (e.g., home visitor qualifications), and processes (e.g., how to refer clients). A matrix was created whereby contract documents comprised the matrix rows and content analysis codes comprised the matrix columns. If the code was not present in the text, the matrix cell was left blank. For contract documents, the level of analysis was the service system and a separate matrix was created for each of the eight systems. The content analysis of contracting documents provided background knowledge for understanding the terminology, references, and examples in the transcripts. In the second analytic stage, transcripts for each service system were analyzed using focused coding around two broad sensitizing concepts: (1) service system influence on organizations through contracts and (2) organizations’ influence on service system through contracts [52]. Coding collectively considered all organizations within a specific service system (rather than between organization analyses across systems). RL coded all transcripts and KF co-coded 20% of the transcripts. Discrepancies and themes in coded material were discussed and resolved between the two coders.

The third analytic stage was a multiple case study analysis. This approach allowed us to understand the complexity of a broader phenomenon (interactions around contracting arrangements) by examining the commonalities within and differences across the unique contexts provided by each case (service system) [53]. Contracting document information and relevant coded transcript material were integrated and organized for each service system. This created eight distinct cases, whereby each system comprised a case. These cases satisfy Stake’s [53] main criteria for case selection in that they are relevant to the phenomenon of interest, are contextually diverse, and provide an opportunity to learn about complexity (i.e., “how the phenomenon performs in different environments”).

Next, patterns and themes across the eight cases were assessed [53]. Through this cross-system comparison process, additional specific codes emerged and were integrated into the codebook. Transcripts and contracting documents were then re-reviewed by RL using focused coding around these specific cross-case themes (see Table 3). Illustrative examples across the cases were extracted for each theme. Final results were reviewed and discussed with members of the parent study research team. Strategies to support rigor included co-coding, peer debriefing, and triangulation of contracting documents and transcripts [54]. A fourth strategy to ensure rigor was the creation of an audit/decision-making trail that documented illustrative quotes and coding decisions [54].

**Table 3:** Codes for focused coding of transcripts

Org dependence on service system	
Ex: referrals	
Insufficient funds force org response	
Ex: cross-training	
Building in implementation supports	
Ex: funding for training	
Contract requirements altering org behavior	
Ex: staffing decisions	
Contracts alter org relationships	
Ex: new subcontracting relationships	
Service system dependence on orgs	
Ex: orgs as SafeCare experts	
Orgs negotiating SafeCare contract details	
Ex: caseload size	
Orgs influencing service system processes	
Ex: referral process	

## Results

### Service system characteristics

Table 4 provides descriptive information for the eight service systems included in this study. This information was drawn from the contracting documents and transcripts: contract type, funding level, stability of contract, rigor of contract oversight, perceptions of CBOs’ ability to influence the contract, and information about academic partnerships.

**Table 4:** Service system characteristics

Service system	Contract type as of 2016	Full costs covered by SC contract	Stability of SC contract	Rigor of SC contract oversight	CBOs able to influence SC contract	Contract stipulates work with academic partners	SC-related academic partnerships
State	Performance-based	Yes	Unstable during major contract change	High	No	Yes	Long term
County 1	Cost reimbursement & performance-based	Yes	Stable	High	Yes	In early contracts	Long term
County 2	Cost reimbursement	Mixed views	Stable	Low	Mixed views	No	Short term
County 3	Cost reimbursement	Mixed views	Stable	High	Yes	No	Short term
County 4	Cost reimbursement	Yes	Stable	Low	Yes	In early contracts	Short term
County 5	Cost reimbursement	No	Unstable	Low	Mixed views	In early contracts	Short term
County 6	Cost reimbursement	No	Stable	Low	Yes	No	Short term
County 7	Cost reimbursement	Yes	Stable	Low	Yes	No	Short term

### Content analysis

Contracts specified SafeCare implementation in the following ways (out of 8 service systems): caseload (*n* = 7), length of service delivery period (*n* = 7) and sessions (*n* = 6), use of other services (*n* = 5), client age (*n* = 8), number of SafeCare positions (*n* = 6), home visitor qualifications (*n* = 6), training details (*n* = 8), coaching details (*n* = 6), referral processes for SafeCare cases (*n* = 8), requirements for tracking and reporting SafeCare program data (*n* = 7), delineation of SafeCare outcomes (e.g., number of families receiving SafeCare, closed cases, completed cases, modules completed, pre-and post-test results for modules) (*n* = 7), formal designation of CBOs as lead agencies (*n* = 3), and explicit mention of SafeCare sustainment (*n* = 4). Table 5 summarizes these findings.

**Table 5:** Content analysis of SafeCare contracting documents by service system

	S*	C1*	C2	C3	C4	C5	C6*	C7*
**Service delivery codes**								
Caseload size	x	x	x	x	x	x		x
Length of service delivery period	x	x	x	x	x	x	x	
Length of sessions		x		x	x	x	x	x
Use of other services	x	x	x	x				x
Client age	x	x	x	x	x	x	x	x
**Staffing codes**								
Specific # of SafeCare home visitors, coaches, or trainers		x	x	x	x	x		x
Home visitor qualifications	x	x		x	x		x	x
SafeCare training	x	x	x	x	x	x	x	x
SafeCare coaching	x	x	x	x	x	x		
**Process codes**								
Referral processes	x	x	x	x	x	x	x	x
Data reporting processes	x	x		x	x	x	x	x
SafeCare outcomes	x	x	x	x	x	x		x
**Other**								
Lead agency designated		x		x		x		
Sustainment explicitly mentioned				x	x	x		x

*Notes*. *S* state, *C1* county 1, *C2* county 2, etc. *SafeCare is embedded within a broader child welfare program, not stand-alone SafeCare contract

### Outer to inner context influence around contracting arrangements

**Fig. 1:**
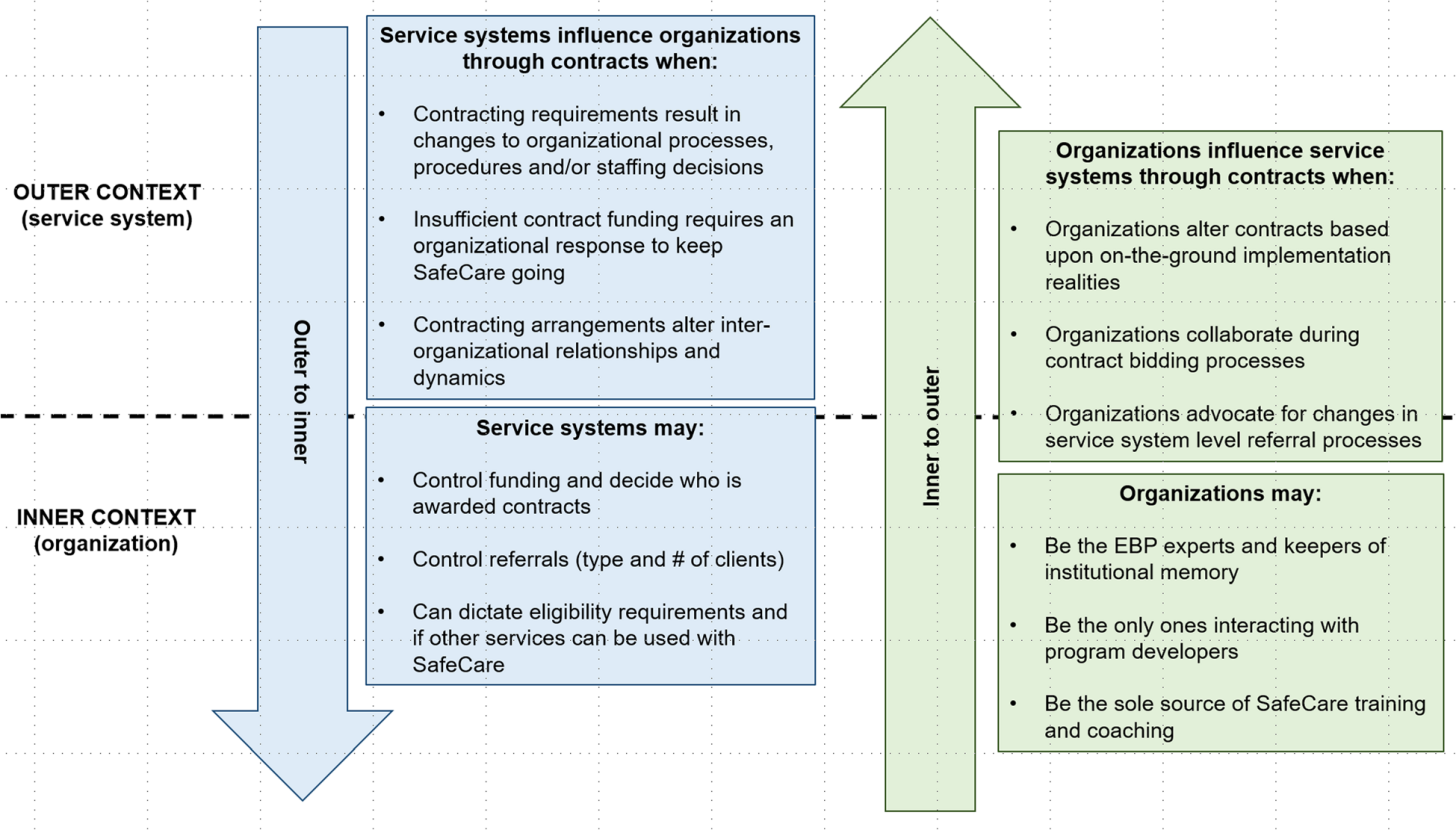
Sources and direction of influence across the outer and inner contexts

SafeCare contracts functioned as a conduit for bi-directional influence across the outer and inner contexts. Outer-inner interactions persisted and were continually negotiated and adapted as SafeCare became sustained in the systems. Figure 1 summarizes these influences. As described in the next sections, the data pointed to several ways that service systems influenced organizations through contracting arrangements. Contracting requirements mandated by the service system typically resulted in changes to organizational-level processes, procedures, and/or staffing decisions. Insufficient contract funding required an organizational response to maintain SafeCare implementation over time. Additionally, contracting arrangements decided at the service system level altered interorganizational relationships and dynamics. Table 6 provides additional context and quotes to illustrate these outer to inner influence context findings.

**Table 6:**
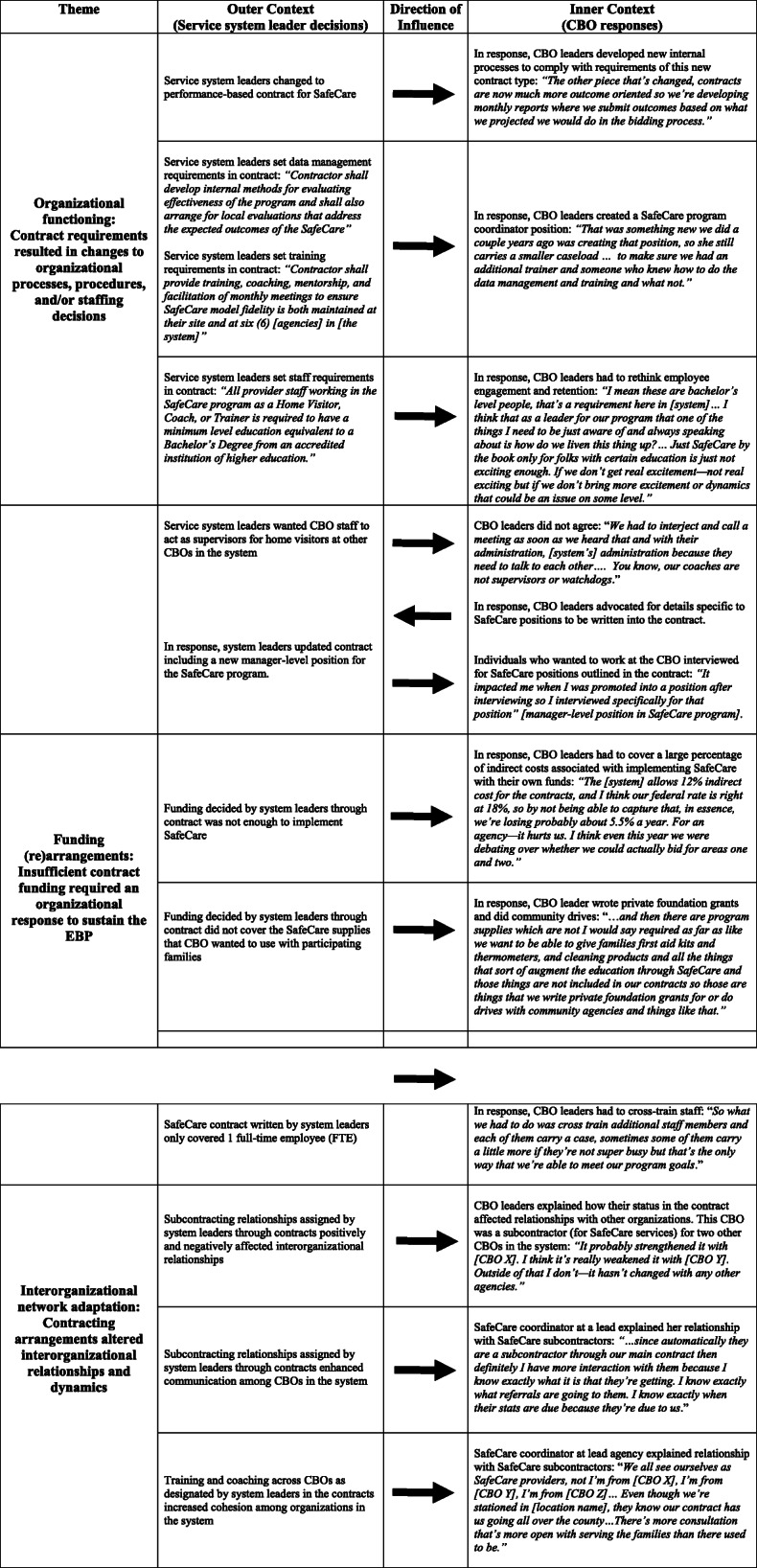
Additional context and quotes for outer to inner context themes

Note: As many details as possible were provided without identifying specific organizations or systems

#### Organizational functioning: contract requirements resulted in changes to organizational processes, procedures, and/or staffing decisions

First, service systems influenced CBOs when contract requirements prompted organizations to change their processes, procedures, and staffing decisions in order to comply. Content analysis illustrated multiple ways that service system decision makers directed SafeCare implementation at the organizational level through contract structure. For example, a contract may have required a specific number of SafeCare home visitors that an organization needed to have, their qualifications, and how they were trained and coached. The contract may have also specified SafeCare itself as the required evidence-based model.

Another example of how service system-level contracts influenced organizations occurred in a service system in which the contract for SafeCare changed from cost-reimbursement to performance-based. As a result, organizations in that system created internal processes to collect and report information required by the service system. An agency leader in this system talked about using the reports generated for contract compliance for internal quality assurance purposes, demonstrating the integration of new contract requirements into organizational improvement processes. In a different service system, the organization designated as a “lead agency” developed a new position to manage the administrative requirements of the contract.

In other service systems, organizations changed hiring processes to interview and promote individuals based on SafeCare roles outlined in the contract, changed employee engagement strategies to comply with a requirement that SafeCare home visitors have at least a Bachelor’s degree, co-located their employees with county staff, and updated organizational procedures to match the contract’s scope of work and to enhance system and organization employee communications to facilitate client referral processes. These examples demonstrated ways in which service systems can influence organizational processes, procedures, staffing decisions, workflow, organizational design, and/or the use of workspace.

#### Funding (re)arrangements: insufficient contract funding required an organizational response to sustain the EBP

A second way that contracting arrangements were a conduit for influence across the outer and inner contexts was related to insufficient funds. Funding decisions at the service system level created implementation barriers or supports. In some systems, SafeCare funding was comprehensive and included costs associated with training, program evaluation, data management, and supplies for families (e.g., electrical outlet covers). In other systems, CBOs had to find ways to make up the difference between what the contract covered and what was actually required to deliver and sustain SafeCare in their communities (including indirect expenses and cost of living increases for staff). As one agency leader stated when discussing the indirect costs that the agency had to cover in order to implement SafeCare under the parameters of its current contract, “We do literally lose money operating the contracts.”

Organizations compensated for insufficient funding in a variety of ways. For example, cross-training staff allowed organizations to fulfill SafeCare obligations and other organizational roles. Other ways that organizations responded to insufficient funding included: fundraising (e.g., securing private donations, writing private foundation grants), pulling from other internal funding sources (e.g., discretionary funds not earmarked for a specific purpose or the agency’s investment income), laying off staff, reducing full-time equivalent staff and the work hours they could devote to SafeCare, and creating a client waitlist. Thus, insufficient contract funding at the service system level influenced organizational level behavior during SafeCare implementation.

#### Interorganizational network adaptation: contracting arrangements altered interorganizational relationships and dynamics

A third way that service systems influenced CBOs through SafeCare contracts was when contract-related decisions altered an organization’s relationship with other organizations in the system. Three of the systems had contracts that formally designated one or more CBOs as a lead agency. Three additional systems had lead agencies not formally designated as such in the contracts. Lead agencies may have had subcontracting relationships with other agencies and served as a local hub for SafeCare training, coaching, and expertise, and this provided pragmatic and economic efficiency compared to relying on the program developer for these functions. Lead agencies may have received more funding to support this larger role. Coordinators at lead agencies traveled to other sites, trained, monitored fidelity and coached staff, and tracked data for employees at other agencies.

Having a contractually dedicated lead agency fostered connections across organizations providing SafeCare. However, non-lead agencies may have been dependent on lead agencies, especially when training new staff. Contract decisions made at the service system level also influenced organizational positions within the network. In one system, an organization transitioned from being a lead agency to a subcontractor. This altered interorganizational dynamics in that, “People are less open in those meetings now than they used to be because one of the agencies is now subcontracting with another” [academic collaborator]. After these contract changes, the agency leader described the situation negatively as having to be “subservient to another agency” and feeling “betrayed by the decision.”

### Inner to outer context influence through contracting arrangements

Referring back to Fig. 1, there were several ways that CBOs influenced service systems through contracting arrangements. Organizations altered contract details based upon on-the-ground implementation realities. Organizations collaborated during contract bidding processes. Additionally, organizations advocated for changes in service system-level referral processes described in the contracts. These examples of inner to outer context influence were evident over time. As SafeCare became sustained, organizations continued to interact with the broader service system in specific ways. Each type is described in detail below. Table 7 provides additional context and quotes for these inner to outer influence context findings.

**Table 7:**
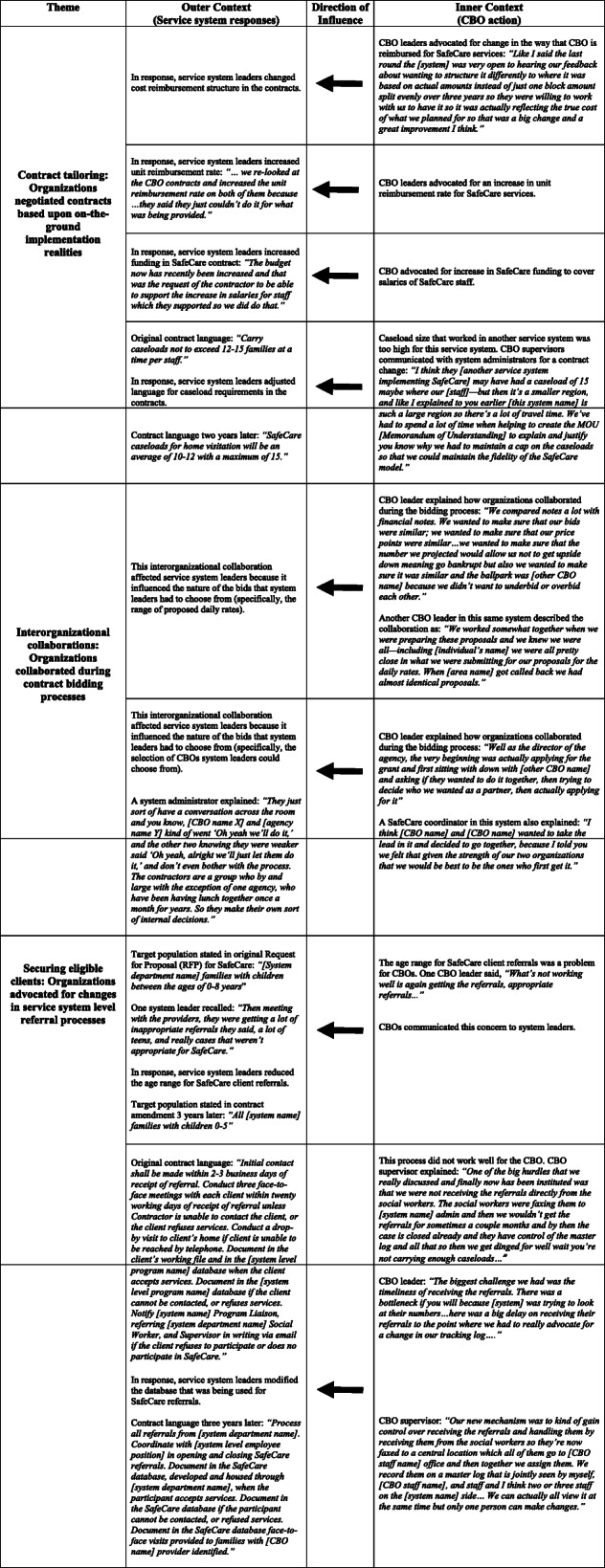
Additional context and quotes for inner to outer context themes

Note: As many details as possible were included without identifying specific organizations or systems

#### Contract tailoring: organizations negotiated contracts based upon on-the-ground implementation realities

First, although service system leaders ultimately decided the terms and structure of contracts, there were multiple examples of CBOs negotiating contract details based on their knowledge of the realities of implementing SafeCare. Organizational leaders negotiated with service system leaders around contract details including the number of client visits, caseload size, and getting paid for “drop-bys” (i.e., visiting referred clients’ homes after a certain number of days if there was no response to other communications). Organizational leaders negotiated other funding-related contract changes including administrative support to comply with changing SafeCare certification standards, additional staff members, increased staff salaries, training costs, and broader operations (e.g., daily rates or unit costs). Other issues that required negotiation included changing the type of contracts and keeping caseloads up to service system standards while also paying for the time that it took home visitors to get SafeCare certified. This required a series of conversations and negotiations between organizational and service system leaders. In sum, CBOs confronted daily realities and challenges of SafeCare implementation and sustainment and used these experiences to influence the service system by negotiating for contract changes.

#### Interorganizational collaborations: organizations collaborated during contract bidding processes

There were clear examples of CBOs collaborating with each other when applying for SafeCare contracts. This was important because such collaboration restricted the number of eligible potential contract awardees from which service system leaders could select. It also influenced the type and nature of bids that service systems received. While individuals were conscientious about avoiding conflicts of interest, they were also frank about informal conversations with each other about subcontracting relationships, proposed rates, and service areas. One service system administrator explained: “…they just sort of have a conversation across the room and you know, [agency name X] and [agency name Y] kind of went ‘Oh yeah we’ll do it.”. A similar situation occurred in another service system. One agency leader explained, “We wanted to make sure that our bids were similar; we wanted to make sure that our price points were similar… we didn’t want to underbid or overbid each other.”

These examples suggest that while service systems had significant influence over contract funding and awardees, organizations also influenced the service system by coordinating with each other around the bidding processes for SafeCare contracts. This illustrates bi-directional processes encompassed in bridging factors as well as the importance of interorganizational networks that are a fundamental construct in the EPIS framework.

#### Securing eligible clients: organizations advocated for changes in service system-level referral processes

A third type of inner to outer context influence was that CBOs advocated for changes in service system-level referral processes. As noted above, service systems can affect an organization’s ability to successfully implement an EBP by setting service delivery standards (e.g., specifying types of clients referred to contracted organizations). In two systems in particular, organizations actively worked to change SafeCare referral processes at the service system level. In one of these systems, organizations worked with government administrators to create a new referral approach that engaged a SafeCare home visitor and a liaison from a different program. To generate referrals, this system also created more stringent screening and instituted more robust referral data tracking (e.g., contract requires quarterly reports including number of referrals, broken out by source and reasons for closing both a referral and a case). An organization’s ability to implement SafeCare was dependent on having enough eligible clients and having referrals waiting as soon as SafeCare training was complete (important for effective learning, transfer of training, and caseworker expertise development). Advocating for changes in the contract’s referral processes was a way that organizations influenced service systems during SafeCare implementation in order to facilitate implementation and sustainment.

### Service systems’ dependencies on contracted organizations

**Fig. 2:**
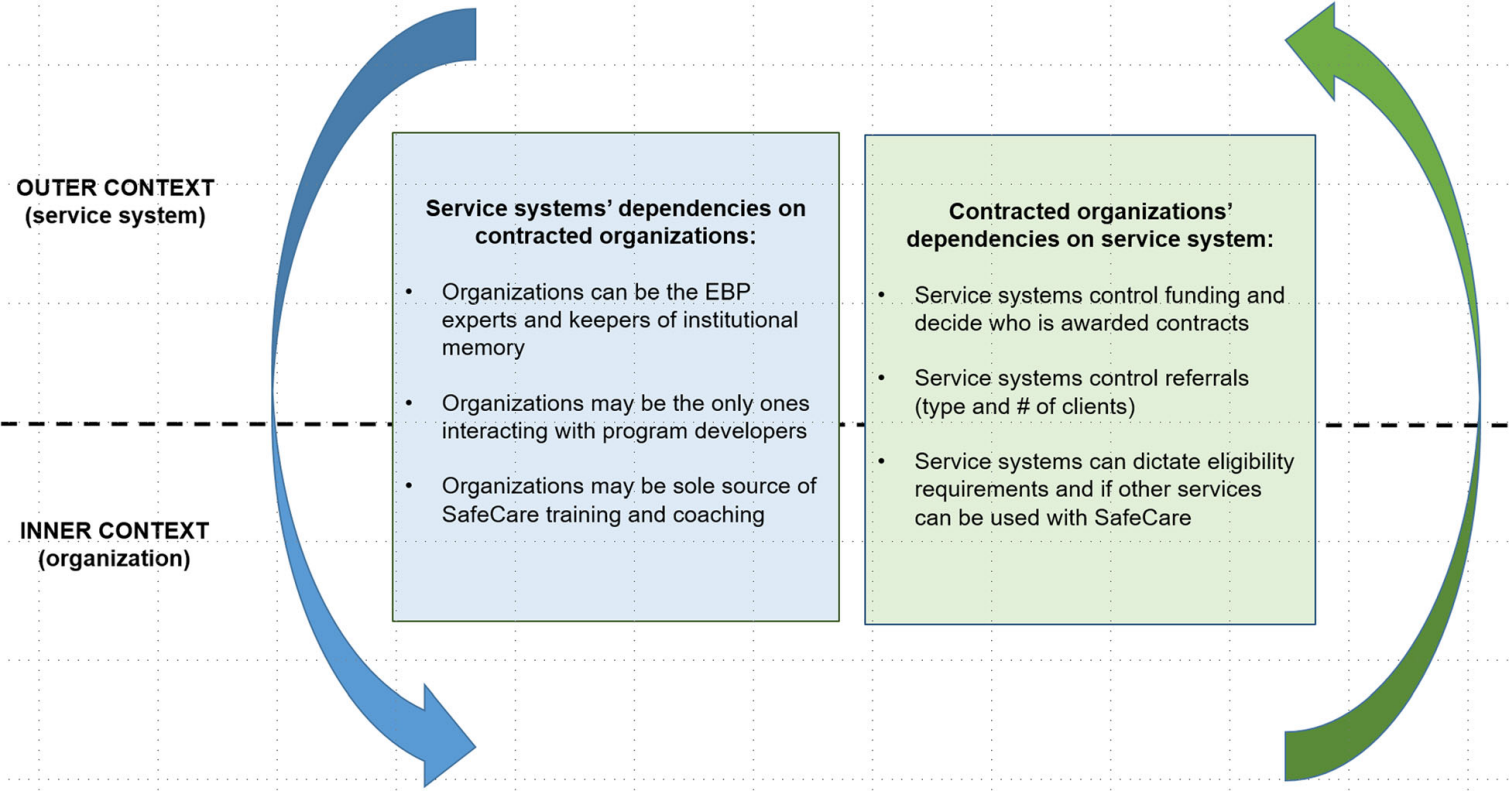
Dependencies across the outer and inner contexts

There were multiple dependencies around contracting arrangements. Figure 2 summarizes these dependencies, and each example is described in greater detail in the next sections. While much of the influence around contracting arrangements was at the service system level, several sources of service system dependence on CBOs emerged in the data. First, because service systems were contracting out SafeCare services, contracted organizations were often the EBP experts and could be keepers of institutional memory when service system level leadership changed. This institutional memory represented knowledge about the history of SafeCare implementation in the system, including initial contract development, contract decision-making, and contract changes over time. As one service system administrator described,

*It’s nice because they can give the history. Sometimes if I’m not sure about something I can call the contractors and say why is the process this way and they’ll give the history of that and I can trust that they’re giving me—because I’ve gone back and looked at past contracts and I’m like, ‘Oh they gave me exactly that.’*

Thus, trust between system and organization leadership was developed in the face of leader turnover. An agency leader in a different system also addressed the need to “educate” the service system when there was a turnover. At times, service system administrators were dependent on organizational level stakeholders to understand SafeCare itself and communicate how it had been implemented in the system over time. “I think as the years have gone on we have probably in the CBOs have become more of the experts in delivering this…we’re relying more on them,” one system leader explained.

Second, service systems were dependent when the organizations were the only ones interacting with program developers. One service system administrator described the lead agency as “bypassing us” when communicating with program developers. An administrator in another service system discussed how the organization’s SafeCare coordinator worked directly with the program developers managing SafeCare fidelity and program data for the whole system. This type of dynamic made service system administrators dependent upon agency leaders to reliably relay information and feedback from the program developers.

Third, service systems were dependent when the contracted organizations were the sole source of SafeCare training and coaching for the entire system. One service system administrator stated: “The CBOs actually are the ones who have the expertise. I think they even train our [staff]...” To summarize, potential sources of service system dependencies on organizations around contracting arrangements were that CBOs were often the EBP experts and keepers of institutional memory, may have been the main link to program developers, and may have been the sole source of program training and coaching for the entire system. These functional dependencies were institutionalized as service systems moved from the implementation to sustainment phases.

### Contracted organizations’ dependencies on service systems

Several sources of organizations’ dependence on service systems were also identified in the data. Most prominent was that service system leaders controlled funding and the system’s procurement and contracting processes influenced decisions regarding which organizations were awarded contracts. Additionally, service system administrators often dictated client eligibility requirements and controlled the number and type of client referrals that organizations received. Eligible client flow subsequently impacted the CBOs’ ability to implement SafeCare. For example, one service system increased the maximum age for SafeCare clients resulting in organizations receiving an influx of clients who were less appropriate for (and therefore less likely to successfully complete) the SafeCare model.

While each organization in this sample provided multiple types of client services, system leaders could also influence whether other services could have been used concurrently with SafeCare. Similar to client flow, this represented a source of organizational dependence because using other services along with SafeCare affected an organization’s ability to engage and retain SafeCare clients. The CBOs were dependent on service systems when contracting arrangements structured service delivery in ways that affected the likelihood of implementation success: funding, client flow, and ability to concurrently use multiple service models. These dependencies remained as organizations moved from initial implementation to sustainment phases.

## Discussion

Outer and inner contexts are key features of the EPIS and other frequently cited implementation frameworks [2–4]. Most implementation research separately examines outer and inner context factors, thereby treating organizations as closed systems. We propose that researchers explore bridging factors that represent the dynamics, interactions, and exchanges that cross the outer-inner context boundaries. Bridging factors are relevant to any implementation framework that distinguishes between an outer and inner context. However, we grounded this work in the EPIS framework, a process and determinant framework [55] that specifically highlights the importance of this concept [1].

Bridging factor research reflects the interdependence of systems and organizations and would benefit from an open-system theoretical perspective. Resource dependence theory is an open-system theory that can enhance our understanding and use of the bridging factor concept. Resource dependence theory posits that organizational survival depends on acquiring and maintaining essential resources and that organizational behavior can be understood by looking at responses to environmental contingencies and constraints [41].

This study explored how contract arrangements can be a bridging factor. Contracting arrangements as a way for public systems to structure and deliver services through non-governmental organizations is not a new topic [27, 31, 32, 39, 40]. What is new is focused attention on the processes (e.g., negotiation, bi-directional influence) that link the outer and inner contexts as systems and organizations implement and sustain an EBP [39]. Rather than separately reporting system administrators’ and organizational leaders’ experiences, our innovative analyses illuminated dynamic outer-inner boundary interactions around a shared experience (in this case, executing contracting arrangements).

For example, organizational issues identified in this study (e.g., changing internal processes to align with contract requirements, finding additional funding to supplement contract inadequacies) illuminated CBO strategies to adjust to environmental constraints imposed by service systems. The bi-directional application of resource dependence theory also highlighted that organizations are not passively controlled and constrained by the environment. Instead, organizations shaped the broader service system during EBP implementation.

For example, organizations influenced the system through contract tailoring, interorganizational collaboration during bidding processes, and system level referral process change. Conversely, contracting requirements mandated by the service system resulted in changes to organizational processes, procedures, and staffing decisions. Insufficient contract funding required organizational responses to make up the difference, and contracting arrangements altered interorganizational relationships.

We expect other types of bi-directional influence to emerge as different bridging factors (e.g., community-academic partnerships, EBP purveyor/intermediaries, or policies that require outer-inner actor interaction and collaboration during EBP implementation) are more closely examined [1]. We also expect to see different types of bi-directional influence under different inner-outer boundary conditions (e.g., hospital units within a regional system, churches within a neighborhood, schools within a school district, or county-run agencies within a state). Articulating this bi-directional influence can help us understand which bridging factors are most relevant to stakeholders and what specific activities within these “bridges” can be intentionally and systematically modified to support system-wide EBP implementation.

This study also identified how resources beyond funding are needed to sustain an EBP [56]. For example, a resource that service systems have and organizations need is eligible clients who can successfully engage in the intervention. This is needed for effective EBP learning, transfer of training, and expertise development. Service system actors may control important aspects of referral processes, such as the number of clients referred, eligibility requirements, and an organization’s ability to engage and retain clients by supplementing SafeCare with other curricula.

More interesting perhaps is that the data also identified resources that fostered service system dependence upon organizations that implement an EBP. Such resources include EBP expertise (including established relationships with and access to program developers), and local and efficient training and coaching capacity [15]. Organizations build these assets over time. While service system leaders shape and carry out contract and procurement processes [39], system-wide sustainment of an EBP depends on the ability of organizations to successfully deliver the practice with fidelity. Again, bridging factors go beyond focusing only on the importance of outer and inner contexts and draw our attention to concrete and modifiable ways that these bi-directional linkages may affect implementation (e.g., managing critical resource dependencies, like client flow).

### Strengths and limitations

This study benefited from a comprehensive longitudinal dataset that included outer and inner context stakeholder perspectives, and triangulated analyses of transcripts and contracting documents. Examining multiple years of contracting documents and transcripts allowed for an examination of interactions, influence, and dependence over time. Key limitations are that the analysis focused on a single EBP and only on sustaining sites. Outer-inner interactions around contracting arrangements could be quite different in the context of another EBP or among systems that failed to successfully implement an EBP.

### Future research

One avenue for future research is exploring how bridging factors affect the selection and tailoring of implementation strategies. Implementation strategies are defined as “a systematic intervention process to adopt and integrate evidence-based health innovations into usual care” [57]. Examining outer-inner influences and resource dependencies can illuminate environmental constraints and available and needed resources (e.g., human capital, social capital, infrastructure) that affect the potential success of implementation strategies in a particular service system [42, 58]. Findings from this study could help leaders who are considering system-wide implementation assess which strategies (e.g., accessing new funding, modifying payment/fee structures, changing accreditation requirements, altering credentialing and licensure standards, developing resource sharing agreements) [59] are most likely to be feasible and sustainable given the structure of and resources included in the contracting arrangements.

This work can also be a starting point for examining how contracting arrangements “bridge” outer and inner contexts in settings outside of the USA [33–38]. Another priority for future research is to identify new bridging factors, such as the specific ways that client-focused advocacy groups link outer and inner contexts during different implementation stages.

Future work may also revisit existing implementation strategies through a bridging factor lens. Some implementation strategies, like the Interagency Collaborative Team (ICT) model, for example, are designed to develop and institutionalize bridging factors [60]. The ICT model engages relevant stakeholders within and across outer and inner contexts. It supports multiple processes for facilitating open communications and collaborations toward an identified implementation goal [60]. Finally, there are many questions related to bridging factor methodology and measurement. One issue is that the outer-inner boundary is context-specific. While this study examined organizational and service system “bridges”, future studies may examine different outer and inner boundaries. This study offers one methodological approach and brings to light issues that researchers may consider including the need for longitudinal data (including documents) and the representation of diverse outer and inner stakeholder perspectives.

## Conclusions

This study deepens our understanding of bridging factors and illuminates a specific methodological approach for studying them. The focus on contracting arrangements grounds the bridging factor concept by showing specific sources of influences, dependence, and diverse resources that are exchanged across the outer-inner boundary as a new practice becomes sustained. Explicit attention to outer-inner influences and resource dependencies can enhance the selection and tailoring of implementation strategies, especially those that require system-wide coordination.

## Data Availability

The datasets generated and/or analyzed during the current study are not publicly available. Questions can be directed to the corresponding author (GAA).

## Abbreviations

CBO: Community-based organization
EBP: Evidence-based practice
EPIS: Exploration-Preparation-Implementation-Sustainment framework
